# Establishment and Application Prospect of Reverse Transcriptase Recombinase-Aided Amplification Assay for Subgroup C Avian Metapneumovirus

**DOI:** 10.3390/vetsci11030122

**Published:** 2024-03-07

**Authors:** Yuhang Bai, Xiuhong Wu, Jiajia Liu, Zhanxin Wang, Mengyue Dong, Tong Li, Zhenkai Dai, Hongxin Li, Qingmei Xie, Xinheng Zhang

**Affiliations:** 1 State Key Laboratory of Swine and Poultry Breeding Industry & Heyuan Branch, Guangdong Provincial Laboratory of Lingnan Modern Agricultural Science and Technology, College of Animal Science, South China Agricultural University, Guangzhou 510642, China; 2 Guangdong Engineering Research Center for Vector Vaccine of Animal Virus, Guangzhou 510642, China; 3 South China Collaborative Innovation Center for Poultry Disease Control and Product Safety, Guangzhou 510642, China; 4 Key Laboratory of Animal Health Aquaculture and Environmental Control, Guangzhou 510642, China; 5 Guangdong Provincial Key Laboratory of AgroAnimal Genomics and Molecular Breeding, College of Animal Science, South China Agricultural University, Guangzhou 510642, China; 6 Guangdong Wen’s Foodstuffs Group Co., Ltd., Yunfu 527439, China

**Keywords:** subgroup C avian metapneumovirus, reverse transcriptase recombinase-aided amplification assay, N gene, constant temperature detection

## Abstract

**Simple Summary:**

The emergence of aMPV-C will have a detrimental impact on egg quality, elevate chicken mortality rates, and severely impede the progress of the chicken industry. Hence, there is an urgent need to establish a rapid and convenient method for aMPV-C detection. Traditional approaches such as PCR, real-time PCR, ELISA, and virus neutralization are associated with expensive equipment requirements and intricate operational procedures that impose certain limitations. In this study, we have developed an RT-RAA method utilizing fluorescent probe technology for isothermal nucleic acid amplification. This novel approach enables the detection of aMPV-C within just 30 min at 41 °C while exhibiting exceptional sensitivity and specificity.

**Abstract:**

Among broilers, the main pathogen that leads to swollen head syndrome (SHS) is the subgroup C avian metapneumovirus (aMPV-C). The aMPV-C infection can lead to an upsurge in the rate of soft-shell eggs, resulting in reduced egg production and seriously affecting the economy of the livestock industry. Therefore, a rapid method for aMPV-C detection needs to be invented. According to the N gene of aMPV-C, we designed the specific probe and primer and created a reverse transcription recombinase-aided amplification assay (RT-RAA) for the detection of aMPV-C. aMPV-C could be detected quickly and specifically by this method at 41 °C for 30 min. The sensitivity assay inferred that the minimum detection threshold of RT-RAA was 3.38 × 10^1^ copies/μL. A specificity assay showed that the RT-RAA method did not cross-react with other subgroups (aMPV-A, aMPV-B, aMPV-D) or other viruses (H9N2, NDV, IBV, IBDV). Forty samples of known clinical background were tested by RT-RAA and RT-qPCR. The two approaches had a 100% correlation rate. In conclusion, this research successfully created an RT-RAA assay for aMPV-C.

## 1. Introduction

Avian metapneumovirus (aMPV), or avian pneumovirus (APV), is also known as turkey rhinotracheitis virus (TRTV) because it can infect turkeys and cause turkey rhinotracheitis (TRT) [1]. In birds, aMPV primarily leads to swollen head syndrome (SHS), as well as acute upper respiratory tract infections. In many foreign countries and regions, aMPV brings economic losses and animal welfare problems second only to the Avian influenza virus (AIV). aMPV infection in laying hens can lead to an increase in the rate of soft-shell eggs, resulting in reduced production of eggs. It is also the main pathogen causing SHS in broilers, which seriously affects the economy of the livestock industry [2].

aMPV belongs to the family Paramyxoviridae and a subfamily called Pneumoviridae. Its genus category is referred to as Metapneumovirus. It is a single-stranded, segmented, negative-strand RNA virus with a genome length that measures approximately 13 kb. The sequence of aMPV gene from 3′ to 5′ is 3′-leader-N-P-M-F-M2-SH-G-L-trailer-5′ [3]. Since the first detection of aMPV in 1978, aMPV has been widely spread around the world, causing severe damage to the world’s poultry industry [4]. aMPV can be divided into four subtypes, which comprise aMPV A-D [5,6]. Two new aMPV subtypes have been found in parakeets and gulls in the United States [7]. More subtypes may emerge in the future. In 1978, aMPV was found in South Africa and has since been widespread worldwide. As of now, aMPV has been found in many areas where poultry is raised in Europe, Asia, South America, Africa, and North America [8,9,10,11,12,13]. It can be seen that aMPV has an extensive spread range. aMPV-A and aMPV-B have been documented in several European countries [14]. aMPV-C exists in France, the United States, and China [15], and aMPV-D is exclusively found in France [16].

Clinical symptoms of aMPV infection usually occur within 2–10 days and are characterized by cough, sneezing, tracheal rales, nasal mucus secretion, inflammation of the conjunctiva, infraorbital sinus swelling, and submandibular edema. aMPV infection also leads to a decrease in the egg production rate of broiler breeders, seriously affecting egg quality, and is accompanied by peritonitis [17]. Since aMPV primarily infects the upper respiratory tract, aMPV can additionally be transmitted through the air, especially through airborne particles [18]. Although some studies have detected aMPV in laying hens’ reproductive tracts and found that the virus may have vertical transmission, it has not been confirmed that vertical transmission is the main transmission route [17,19]. In addition, the transmission of the aMPV is also related to the migration of wild birds, the movement of people and vehicles, the transportation of infected poultry, contaminated water sources, and feeding tools.

aMPV has been widely present in China. In 2008, 107 organs or swabs from chickens were collected and tested in live poultry markets in southeast China, and aMPV-A was found [20]. In 2012, aMPV-C was effectively extracted from chickens with severe respiratory symptoms on broiler farms in southeastern China [21]. In 2016, researchers collected turbinate bone samples from chickens with SHS on a farm in Liaoning Province, China, and successfully isolated an aMPV-B strain through the Vero cell subculture [22]. So far, there have been no reports of aMPV-D in China.

Current methods for aMPV detection can be divided into molecular detection and serological detection, including conventional PCR, real-time fluorescence quantitative PCR (qPCR), enzyme-linked immunosorbent assay (ELISA), and virus neutralization (VN) [7]. Traditional molecular detection methods encompassing conventional PCR and qPCR have high specificity and sensitivity, but require expensive and complex machinery and experienced laboratory personnel [23,24]. Compared with molecular detection, serological detection is conducive to the large-scale screening and monitoring of livestock and poultry. ELISA is currently the most commonly used technique [25], and most tools have been developed into ELISA kits. However, the antigenicity differences between the antigens used can lead to differences in the test results’ sensitivity and specificity [26,27]; the early diagnosis of aMPV infection may be affected by inappropriate ELISA antigen selection. Virus neutralization tests can be carried out in many systems. For example, when using TOC to conduct a VN test, the movement of the cilia is used as the judgment index, and it usually takes about 10 days to for the result of this method to be obtained. And VN tests are expensive and time-consuming [7]. Therefore, there is a need for a sensitive and rapid diagnostic method to detect and control aMPV infection.

The reverse transcription recombinase-aided amplification (RT-RAA) assay is a recent approach that utilizes isothermal amplification. The assay technique does not demand a complicated temperature setup, and solely requires exponential DNA amplification that ranges from 20–30 min at a sustained temperature of 37 °C to 42 °C [28]. DNA polymerase, recombinases, and single-stranded DNA-binding proteins are the primary basic enzymes involved in RAA’s reaction process [29]. RT-RAA can directly use RNA as a template [30]; it can be combined with a fluorescent probe system and then detected using conventional laboratory instruments. It can also be visualized using lateral flow strips (LFS) and portable blue light instruments [29,31,32], so it is more suitable for field diagnostic applications. In general, the benefits of RT-RAA comprise high specificity as well as high sensitivity, a short detection time, and no need for expensive equipment. So far, this method has been used in animal and plant quarantine and environmental monitoring [33,34,35].

There are eight main structural proteins in aMPV-C. These are nuclear protein (N), phosphoprotein (P), fusion protein (F), second matrix protein (M2), small hydrophobic protein (SH), matrix protein (M), surface glycoprotein (G), and RNA polymerase (L).

Through the alignment of the nucleotides sequences of the N, P, M, F, and G proteins, researchers have found that aMPV-A, aMPV-B, and aMPV-D are more homologous than aMPV-C [36]. Moreover, some studies have shown that subgroup C strains are more virulent than subgroup A and B strains [15]. However, there is no rapid isothermal detection method for aMPV-C in China at present.

We designed probes and primers as per the N gene sequence of aMPV-C because the N protein gene sequence is highly conserved, and we created an RT-RAA isothermal amplification detection approach for aMPV-C in this study. This method can detect aMPV-C within 30 min, with high specificity and sensitivity, and requires only cheap equipment. It may become a key factor in the prevention of aMPV-C in the future.

## 2. Materials and Methods

### 2.1. Viruses and Clinical Samples

We maintained the H9N2 subtype avian influenza virus (H9N2), Newcastle disease virus (NDV), infectious bursal disease virus (IBDV), and infectious bronchitis virus (IBV) in our laboratory. Wen’s Foodstuff Group Co., Ltd. (Yunfu, China) contributed to the subgroup C avian metapneumovirus (aMPV-C), subgroup A avian metapneumovirus (aMPV-A), subgroup B avian metapneumovirus (aMPV-B), and subgroup D avian metapneumovirus (aMPV-D). Furthermore, they additionally donated 40 chicken lung tissue samples of known clinical background (Yunfu, China), including 10 positive and 30 negative samples. All of the above samples were extracted as stipulated by the instructions of the extraction kit for nucleic acid from viruses (Vazyme, Nanjing, China). An ultra-low-temperature freezer was utilized to store the recovered nucleic acids at −80 °C.

### 2.2. Primers and Probe

**Figure 1:**
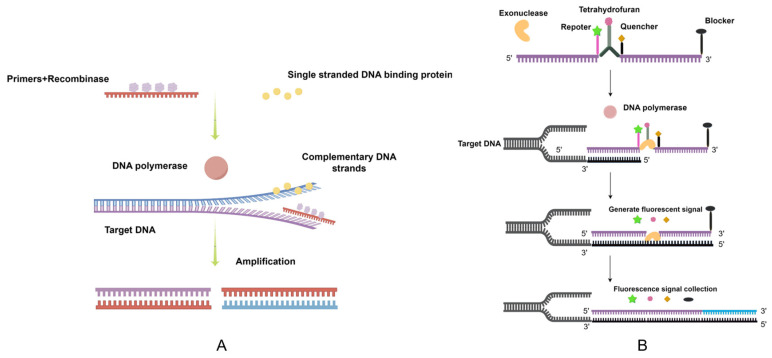
Schematic diagram of the RT-RAA reaction process. (**A**) Mechanism of RT-RAA reaction. (**B**) Principle of fluorescent RT-RAA. We drew this schematic diagram using Figdraw. Different colors are necessary to more intuitively express the process of complementary pairing of DNA double strands.

**Figure 2:**
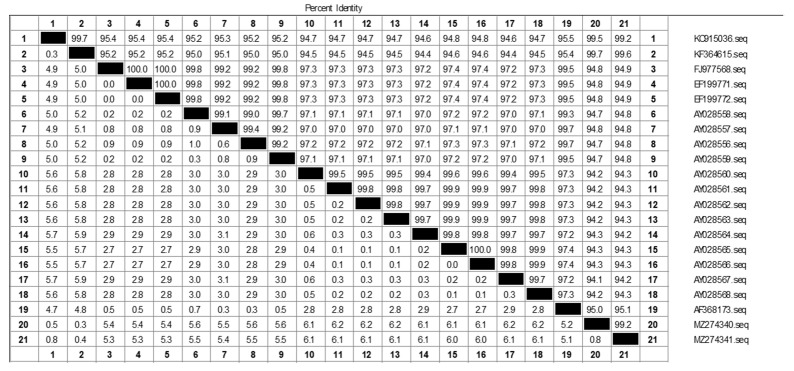
Percent identity and divergence score matrix of the N gene of aMPV-C. Identies of at least 94% indicate that the N gene is highly conserved.

A schematic diagram of RT-RAA is shown in (Figure 1). According to aMPV-C sequences (GenBank accession number) KC915036, KF364615, FJ977568, EF199771, EF199772, AY028558, AY028557, AY028556, AY028559, AY028560, AY028561, AY028562, AY028563, AY028564, AY028565, AY028566, AY028567, AY028568, AF368173, MZ274340, and MZ274341), we used DNAMAN (Lynnon Biosoft, version: v9.0., California, USA)and DNASTAR (DNASTAR, Inc., Madison, WI, USA) for multiple sequence analysis alignment, and found that the N gene of aMPV-C had high homology (Figure 2). Therefore, RT-RAA probes and primers were designed according to the conserved region of the N gene (Table 1). At the same time, we also designed RT-qPCR primers for the subtype C of the N gene. Sangon Biotech (Shanghai, China) was responsible for developing the primers and probes.

### 2.3. Plasmid Standard Generation

Depending on the N gene of aMPV-C (GenBank: MZ274341), we designed a pair of primers and amplified the product via PCR. The length of the product was 515 bp, and it was cloned into the PMD-19T vector (Takara, Dalian, China). The ligation products were converted into DH5α (Vazyme, Nanjing, China) competent cells and plated on AMP-resistant AGAR plates at 37 °C overnight. Onto AMP-resistant LB medium, the single colonies were plated and cultured at 37 °C. The plasmid was drawn out from the bacterial solution and sequenced, and the correctly sequenced plasmid was used as the standard. Upon a successful plasmid construction, the concentration of plasmid was determined to be about 127.624 ng/μL. The plasmid was calculated to be about 3.38 × 10^10^ copies/μL.

### 2.4. Validation of RT-RAA Primers

To validate the size of product fragment by the RT-RAA primer, we adopted aMPV-C nucleic acid and RNase-free ddH_2_O as positive and negative controls, correspondingly. The RT-PCR system comprised RNase-free ddH_2_O 6.0 µL, RT-RAA-Forward/Reverse primer 1.0 µL, One Step Enzyme Mix 1 µL, RNA 1 µL, and 2 × One Step Mix 10.0 µL (Vazyme, Nanjing, China). Program was first heated at 50 °C for 30 min, followed by predenaturation at 95 °C for 3 min, denaturation at 95 °C for 10 s, and annealing at 57 °C for 30 s for a total of 35 cycles, as well as extension at 72 °C for 4 min. A 1% agarose gel was involved in examining the RT-PCR products.

### 2.5. RT-RAA Reaction System Development

The RT-RAA reaction system is elucidated below. The total system was 50 μL, incorporating one tube of RT-RAA reaction dry powder, 2.5 μL of B buffer, 25 μL of A buffer from RT-fluorescence nucleic acid amplification reagent (Zhuangbo, Nanning, China), 2 μL of forward primer, 0.6 μL of probe, 2 μL of reverse primer, 12.9 μL of ddH_2_O, and 5 μL of template. The reaction tube of the prepared RT-RAA reaction system was turned upside down 6 to 8 times, and the reaction solution was thoroughly mixed and centrifuged at 800 rpm for 15 s to centrifuge all of the reaction solution to the bottom of the tube. The reaction solution was quickly placed into a fluorescence quantitative PCR instrument, and the reaction program consisted of 41 °C for 60 s, 41 °C for 30 s, and 40 cycles.

### 2.6. Assay for RT-RAA Sensitivity

The prepared aMPV-C N standard plasmid was serially diluted 10-fold from 10^5^ copies/μL to 10^1^ copies/μL. Each concentration of 5 μL was added to the RT-RAA reaction system described above for amplification.

### 2.7. Assay for RT-RAA Specificity

Using aMPV-C nucleic acid and ddH_2_O as a positive control negative control, correspondingly, 5 μL samples of NDV, IBV, IBDV, H9N2, aMPV-A, aMPV-B, and aMPV-D were used as templates for specificity analysis by placing them into the RT-RAA reaction system described above for amplification.

### 2.8. Assay for the RT-RAA Repeatability

We used 10^3^ copies/μL to 10^1^ copies/μL of plasmid as a template. The reaction template utilized 5 μL for each concentration. This was repeated three times for repeatability analysis, and the samples were placed into the RT-RAA reaction system described above for amplification.

### 2.9. Assessment of Clinical Samples Utilizing the RT-RAA Assay

We used RT-qPCR in conjunction with RT-RAA for nucleic acid detection of 40 samples with known clinical backgrounds. Both results were compared and analyzed.

## 3. Results

### 3.1. RT-RAA Primer Validation

**Figure 3:**
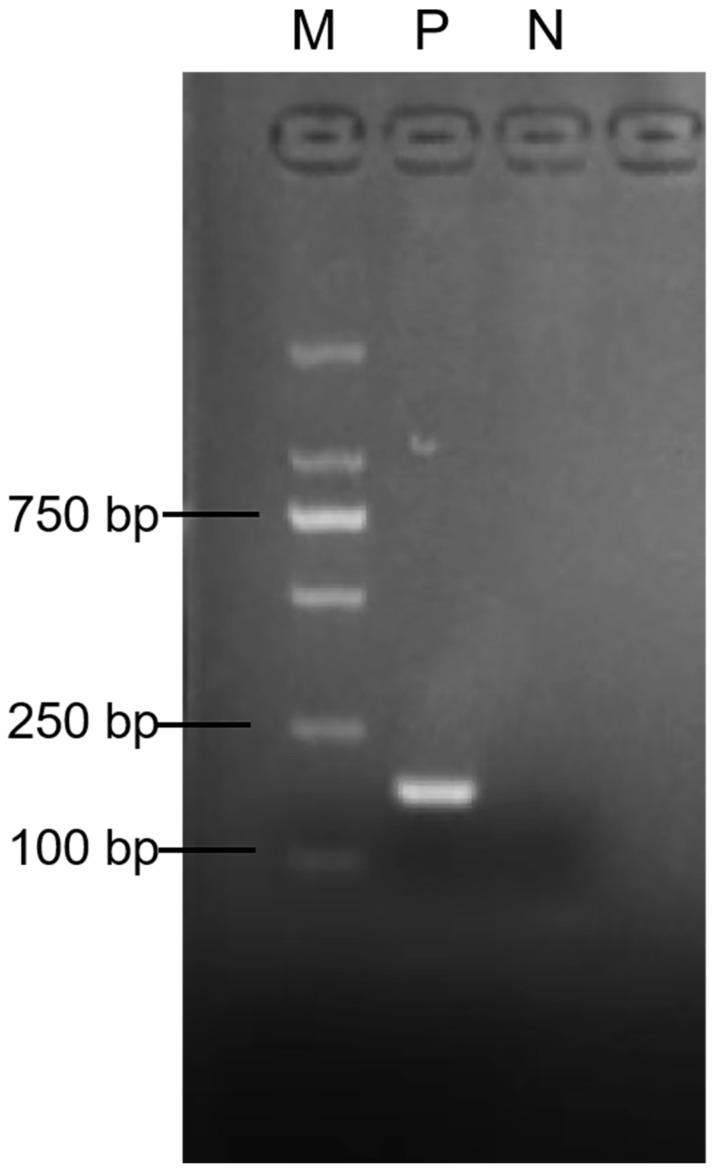
The results of RT-RAA primer size were verified via agarose gel electrophoresis PCR. M: D2000 marker; P: positive control, aMPV-C nucleic acid; N: negative control, ddH_2_O. (Figure S1).

The RT-RAA primers were amplified by PCR experiments, and the results are visualized in Figure 3. It was ascertained that the positive control product was a specific band without the production of a primer dimer, with a 175 bp length consistent with our expected size. It was affirmed that the RT-RAA primers could accurately identify aMPV-C.

### 3.2. The RT-RAA Sensitivity Analysis

**Figure 4:**
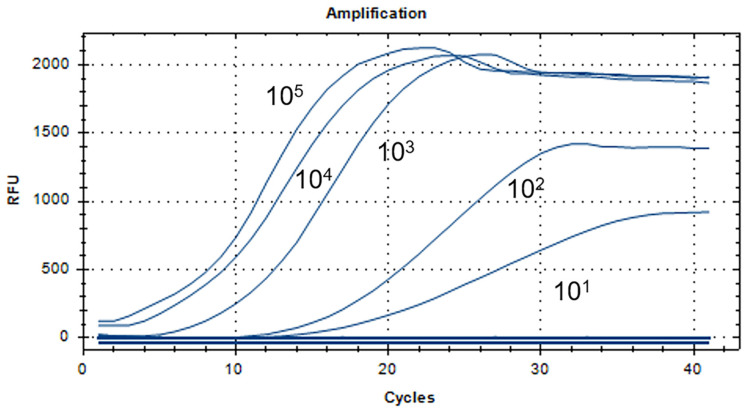
Sensitivity analysis of the RT-RAA assay. The dilution range of the aMPV-C N gene plasmid was 10^1^ to 10^5^ copies/µL, and the minimum detection standard was 10^1^ copies/µL.

The sensitivity analysis results of aMPV-C RT-RAA detection are displayed in Figure 4. The fluorescent signal was still detectable at 10^1^ copies/μL. Therefore, the minimum detection standard of the RT-RAA method was 10^1^ copies/µL.

### 3.3. The RT-RAA Specificity Analysis

**Figure 5:**
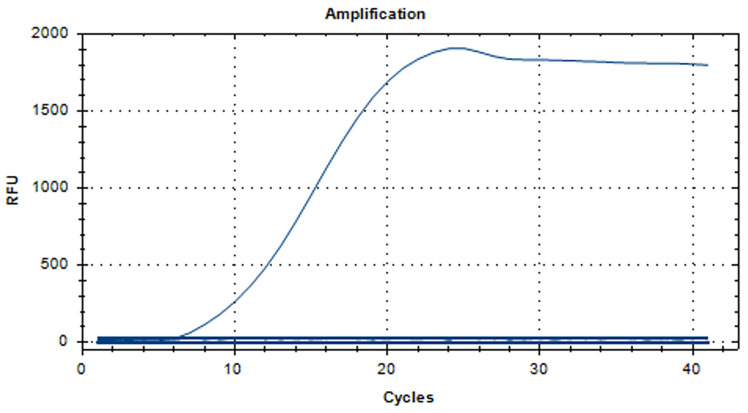
Specificity analysis of the RT-RAA assay. The RT-RAA assay could specifically detect aMPV-C nucleic acid, but had no cross-reactivity with H9N2, NDV, IBV, IBDV, aMPV-A, aMPV-B, or aMPV-D nucleic acid.

The results of the aMPV-C RT-RAA assay analysis are indicated in Figure 5. The positive control exhibited the generation of fluorescent signals, whereas the negative control and other viruses failed to generate the fluorescent signal. Thus, the developed RT-RAA assay could accurately distinguish H9N2, NDV, IBV, IBDV, aMPV-A, aMPV-B, and aMPV-D, and specifically detect aMPV-C.

### 3.4. Repeatability of the RT-RAA

**Figure 6:**
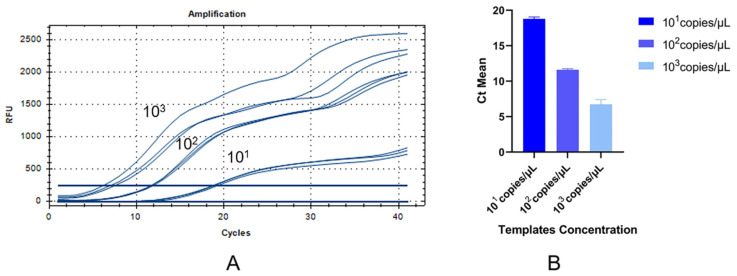
Repeatability analysis of RT-RAA assay. (**A**) The RT-RAA assay was repeated three times with 10^3^ copies/µL, 10^2^ copies/µL, and 10^1^ copies/µL. (**B**) Data from three runs of plasmid standards to analyze RT-RAA assay.

The reproducibility analysis of the aMPV-C RT-RAA assay is shown in Figure 6. They implied that the peak time of the amplification reaction occurred at 10^3^ copies/µL, 10^2^ copies/µL, and 10^1^ copies/µL, repeated three times; the curve shape was similar; and no fluorescence signal was generated in the negative control. Table 2 exhibits the coefficients of variation (CVs) of 9.79%, 1.21%, and 1.44%, respectively, which were all within 10%. This shows the great repeatability of our RT-RAA method.

### 3.5. RT-qPCR Standard and Melting Curves for aMPV-C

**Figure 7:**
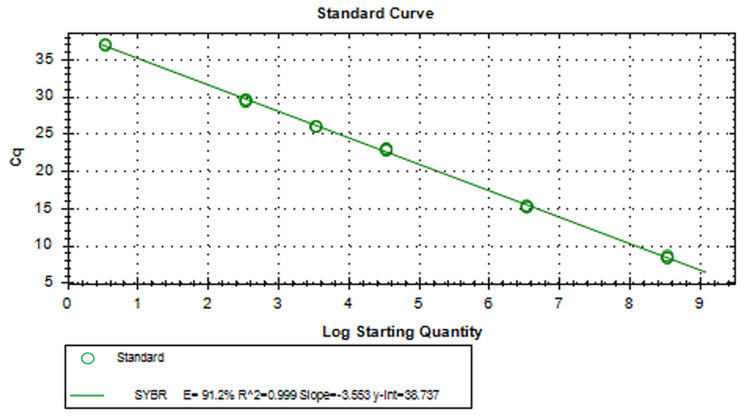
RT-qPCR standard curves for aMPV-C. The correlation coefficient of R^2^ value of the standard curve was 0.999, and the amplification efficiency was 91.2%.

**Figure 8:**
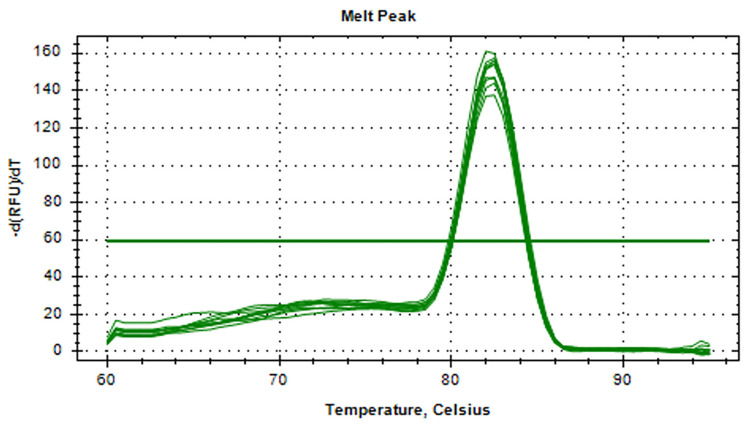
RT-qPCR melting curves for aMPV-C. The melt temperature of RT-qPCR for aMPV-C was 82.5 °C.

Figure 7 presents the standard curve, with 0.999 as the correlation coefficient of R^2^ values and an amplification efficiency of 91.2%. As shown in Figure 8, the RT-qPCR melting curve of aMPV-C showed a single peak and no miscellaneous peaks, with a melting temperature of 82.5 °C.

### 3.6. Evaluation of Testing of Clinical Samples

The detection findings of clinical samples are shown in Table 3. We tested 40 samples of known clinical background with RT-RAA and RT-qPCR, respectively, and both approaches yielded consistent positive as well as negative results. In contrast to RT-qPCR, the time required for RT-RAA was only 30 min, which indicates that RT-RAA detection can yield accurate results quickly and does not require heavy instruments, which can save more time.

## 4. Discussion

Following the outbreak of aMPV, significant losses have been incurred in the poultry industry worldwide. At present, no effective drug has been developed for the treatment of aMPV. Appropriate use of antibiotics can reduce the risk of secondary infection caused by pathogens such as Escherichia coli, Pseudomonas aeruginosa, and Brucella, and can reduce morbidity and mortality. In the selection of antibiotics, we mainly rely on past work experience, possible secondary infection pathogens, and comprehensive resistance monitoring of the farm to select the appropriate antibiotics. Therefore, the most important aspect of the occurrence of aMPV is prevention. At present, there are two most effective prevention options. The first is the reasonable management plan and strict biosafety measures. The staff should clean the feces and urine sewage in the poultry house in a timely manner, do a good job with house ventilation, and have reasonable control of the ambient temperature and humidity. The feeding density should be adjusted according to the different growth stages of poultry, and the personnel, equipment, and feeding tools that come into contact with poultry should be sterilized regularly. The second is vaccination. The use of inactivated aMPV vaccines is widely prevalent in layer and breeder flocks to enhance immunity, as they generate highly effective and durable antibodies. Extensive research has demonstrated that immunization with both aMPV-A and aMPV-B vaccines not only provides robust cross-protection, but also confers defense against aMPV-C [37]. These inactivated aMPV vaccines have gained international market approval and are extensively employed within the poultry breeding industry. The aMPV live attenuated vaccine has been widely used in many countries, including spraying, drinking water, intranasal, and intraocular immunization. At present, the common aMPV-attenuated live vaccines mainly include the low-temperature adapted strain and the Vero cell passaged attenuated strain. The attenuated vaccine can not only stimulate local immunity in the respiratory tract, but also stimulate systemic immunity [25]. With the rapid advancement of modern biotechnology, there has been a growing focus on the development of genetically engineered vaccines. Naylor et al. utilized reverse genetic technology to perform point mutations on the aMPV-A fusion protein F and discovered that this mutated virus not only increased virulence, but also improved immune protection ability, thereby enhancing immunogenicity [38]. Subsequently, Hu et al. employed the LaSota strain of NDV as a vector to insert aMPV-C F and G proteins into the NDV genome, successfully constructing recombinant NDV expressing aMPV-C F and G proteins, which provided complete protection against NDV infection while also offering some degree of immune protection against aMPV infection [39]. At present, live and inactivated vaccines of the aMPV-A and aMPV-B subtypes have been widely used worldwide, and an attenuated vaccine against aMPV-C has been used in the United States [40,41,42]. Since its first identification in China in 2012, aMPV-C has been detected in several provinces [43]. However, the aMPV-C vaccine has not been used in China, so it is urgent to create a simple technique that is both rapid and low-cost to assist in the detection and prevention of the occurrence of aMPV-C.

In this research, we use RT-RAA to generate a convenient and fast method to detect aMPV-C. RAA is an emerging assay that is capable of isothermal amplification of nucleic acids. There are three sets of proteins involved in the amplification of RAA: recombinase, single-stranded DNA-binding protein, and DNA polymerase [29]. The recombinase binds to the primer, forming a complex, which can, at a constant temperature, locate a particular binding site on the target DNA. The double strands of template DNA are split open by single-stranded binding proteins, forming a single-stranded molecule. The primer then searches for complementary bases on the template DNA using DNA polymerase to form a new DNA duplex, and this process is repeated several times to complete the amplification [44]. RT-RAA can directly amplify RNA templates, saving time and costs [45]. After the amplification, special probes are used to detect the target DNA via real-time fluorescence. The probe contains a tetrahydrofuran (THF) site flanked by a fluorophore and a quencher, and a blocker is placed at the 3’ end of the probe. Exonuclease inactivation results in probe stability, the fluorescence is absorbed by the quencher, and no fluorescent signal is emitted. When the probe attaches to the target DNA, the exonuclease is activated. The THF site is recognized and cleaved. Then, the fluorophore is released. When the probe is further amplified to the 3’ end, the blocker is terminated and the probe continues to amplify up to the 5’ end of the target DNA. Therefore, fluorescence signals were detected [46].

According to the N gene of aMPV-C, we designed the specific probe and primer. The RT-RAA assay was proved to be specific for aMPV-C without cross-reaction with other subgroups (aMPV-A, aMPV-B, and aMPV-D) or other viruses (H9N2, NDV, IBV, and IBDV). Current detection methods for aMPV include conventional PCR, RT-qPCR, ELISA, and virus neutralization [23,47,48]. Compared with traditional PCR detection, RT-RAA does not require a gel electrophoresis test and can be completed within 30 min, which saves more time. The lowest detection standard for aMPV-C by RT-qPCR was 10^3^ copies/μL [24]. In our study, we yielded it as 10^1^ copies/μL, which is 100 times more sensitive in contrast to RT-qPCR, and the amplification reaction can be carried out at a constant temperature of 41 °C without complicated temperature change procedures and equipment. Antigenic differences between the antigens used in ELISA methods can lead to differences in the sensitivity and specificity of test results [26,27]. Moreover, ELISA is time-consuming and not suitable for rapid detection. The VN experiment is based on the movement of cilia, and it generally takes about 10 days to obtain the result. However, aMPV-C does not cause ciliary movement arrest [49], so this method is unsuitable for aMPV-C detection.

This study utilized RT-qPCR as well as RT-RAA to analyze the established RT-RAA method. Forty samples with known clinical backgrounds were tested, including ten positive and thirty negative samples. The two methods exhibited a coincidence rate of 100%. Using 10^1^ copies/μL, 10^2^ copies/μL, and 10^3^ copies/μL of standard plasmids to repeat the experiment thrice, the CV of CT values were 1.44%, 1.21%, and 9.79%, respectively, which indicates that the technique developed in this study had good sensitivity and repeatability.

## 5. Conclusions

We successfully developed the RT-RAA assay, which can specifically identify aMPV-C within 30 min with good reproducibility and sensitivity. Additionally, testing can be accomplished quickly, with simple equipment. Our established RT-RAA shows remarkable value for the prevention and control of aMPV-C.

## Figures and Tables

**Table 1:** Primers and probe sequences used in the study.

Primer	Sequence (5′-3′)	Localization	Size of Product (bp)
RAA-F	CTATGCGGAGAGATACTGTATGCCAAGC	139–166	175
RAA-R	TTTGCCTTTACCAAGGGAGTATGTCTTG	285–313	
RAA-P	CCACATTGGGAGCACGGCGTACACAGCGAGA[FAM-dT][THF]C[BHQ-dT]AAAGAACTCAGGTAG-[C3Spacer]		
qPCR-F	TGCGGAGAGATACTGTATGCCAAG	141–165	141
qPCR-R	ATACTGCCTGCACTTCACTACCTG	257–281	
PCR-F	TTCAGGGGATTCAGCTTAGTGAC	8–30	515
PCR-R	GATGCCAGCTTCGTGAAGATT	502–522	

**Table 2:** Analysis of reproducible data for RT-RAA assays.

Templates (Copies/µL)	Average ± SD	CV%
10^1^	18.79 ± 0.27	1.44%
10^2^	11.60 ± 0.14	1.21%
10^3^	6.74 ± 0.66	9.79%

**Table 3:** Comparison of the results of RT-RAA assay and RT-qPCR method for clinical samples.

Sample	Known	RT-RAA	RT-qPCR	Coincidence Rate (%)
Positive	10	10	10	100
Negative	30	30	30	100
Total	40	40	40	-

## Data Availability

All data generated or analyzed during this study are included within the article.

## Supplementary Materials

The following supporting information can be downloaded at: https://www.mdpi.com/article/10.3390/vetsci11030122/s1, Figure S1: Original RT-RAA primer size verification gel electrophoresis.

## References

[1] Franzo G., Tucciarone C.M., Enache M., Bejan V., Ramon G., Koutoulis K.C., Cecchinato M. (2017). First Report of Avian Metapneumovirus Subtype B Field Strain in a Romanian Broiler Flock during an Outbreak of Respiratory Disease. Avian Dis..

[2] Kwon J.-S., Lee H.-J., Jeong S.-H., Park J.-Y., Hong Y.-H., Lee Y.-J., Youn H.-S., Lee D.-W., Do S.-H., Park S.-Y. (2010). Isolation and Characterization of Avian Metapneumovirus from Chickens in Korea. J. Vet. Sci..

[3] Easton A.J., Domachowske J.B., Rosenberg H.F. (2004). Animal Pneumoviruses: Molecular Genetics and Pathogenesis. Clin. Microbiol. Rev..

[4] Buys S.B., du Preez J.H., Els H.J. (1989). The Isolation and Attenuation of a Virus Causing Rhinotracheitis in Turkeys in South Africa. Onderstepoort J. Vet. Res..

[5] Awad F., Forrester A., Baylis M., Lemiere S., Jones R., Ganapathy K. (2015). Immune Responses and Interactions Following Simultaneous Application of Live Newcastle Disease, Infectious Bronchitis and Avian Metapneumovirus Vaccines in Specific-Pathogen-Free Chicks. Res. Vet. Sci..

[6] Rizotto L.S., Scagion G.P., Cardoso T.C., Simão R.M., Caserta L.C., Benassi J.C., Keid L.B., Oliveira T.M.F.D.S., Soares R.M., Arns C.W. (2017). Complete Genome Sequence of an Avian Metapneumovirus Subtype A Strain Isolated from Chicken (*Gallus gallus*) in Brazil. Genome Announc..

[7] Kaboudi K., Lachheb J. (2021). Avian Metapneumovirus Infection in Turkeys: A Review on Turkey Rhinotracheitis. J. Appl. Poult. Res..

[8] Shin H.J., Rajashekara G., Jirjis F.F., Shaw D.P., Goyal S.M., Halvorson D.A., Nagaraja K.V. (2000). Specific Detection of Avian Pneumovirus (APV) US Isolates by RT-PCR. Arch. Virol..

[9] Bennett R.S., LaRue R., Shaw D., Yu Q., Nagaraja K.V., Halvorson D.A., Njenga M.K. (2005). A Wild Goose Metapneumovirus Containing a Large Attachment Glycoprotein Is Avirulent but Immunoprotective in Domestic Turkeys. J. Virol..

[10] Nguyen V.-G., Chung H.-C., Do H.-Q., Nguyen T.-T., Cao T.-B.-P., Truong H.-T., Mai T.-N., Le T.-T., Nguyen T.-H., Le T.-L. (2021). Serological and Molecular Characterization of Avian Metapneumovirus in Chickens in Northern Vietnam. Vet. Sci..

[11] Al-Hasan B.A., Alhatami A.O., Abdulwahab H.M., Bustani G.S., Hameed M.A., Jawad A.H. (2022). First Report of Avian Metapneumovirus Type B in Iraqi Broiler Flocks with Swollen Head Syndrome. Vet. World.

[12] Habte T., Gerber P.F., Ibrahim F., Groves P.J., Walkden-Brown S.W. (2022). Seroprevalence of Major Respiratory Diseases of Chickens in Central Ethiopia in Different Chicken Production Systems. Poult. Sci..

[13] Jesse S.T., Ribó-Molina P., Jo W.K., Rautenschlein S., Vuong O., Fouchier R.A.M., Ludlow M., Osterhaus A.D.M.E. (2022). Molecular Characterization of Avian Metapneumovirus Subtype C Detected in Wild Mallards (*Anas platyrhynchos*) in The Netherlands. Transbound. Emerg. Dis..

[14] Bao Y., Yu M., Liu P., Hou F., Muhammad F., Wang Z., Li X., Zhang Z., Wang S., Chen Y. (2020). Novel Inactivated Subtype B Avian Metapneumovirus Vaccine Induced Humoral and Cellular Immune Responses. Vaccines.

[15] Wang J., Hou L., Wei L., Yan X., Zhu S., Quan R., Li Z., Wang D., Jiang H., Song J. (2023). Characterization of Avain Metapneumovirus Subgroup C Isolated from Chickens in Beijing, China. Poult. Sci..

[16] Brown P.A., Bonci M., Ricchizzi E., Jones R.C., Naylor C.J. (2009). Identification of Two Regions within the Subtype A Avian Metapneumovirus Fusion Protein (Amino Acids 211–310 and 336–479) Recognized by Neutralizing Antibodies. Virus Res..

[17] Jones R.C., Williams R.A., Baxter-Jones C., Savage C.E., Wilding G.P. (1988). Experimental Infection of Laying Turkeys with Rhinotracheitis Virus: Distribution of Virus in the Tissues and Serological Response. Avian Pathol..

[18] Biacchesi S., Skiadopoulos M.H., Yang L., Lamirande E.W., Tran K.C., Murphy B.R., Collins P.L., Buchholz U.J. (2004). Recombinant Human Metapneumovirus Lacking the Small Hydrophobic SH and/or Attachment G Glycoprotein: Deletion of G Yields a Promising Vaccine Candidate. J. Virol..

[19] Khehra R.S., Jones R.C. (1999). In Vitro and In Vivo Studies on the Pathogenicity of Avian Pneumovirus for the Chicken Oviduct. Avian Pathol..

[20] Owoade A.A., Ducatez M.F., Hübschen J.M., Sausy A., Chen H., Guan Y., Muller C.P. (2008). Avian Metapneumovirus Subtype A in China and Subtypes A and B in Nigeria. Avian Dis..

[21] Wei L., Zhu S., Yan X., Wang J., Zhang C., Liu S., She R., Hu F., Quan R., Liu J. (2013). Avian Metapneumovirus Subgroup C Infection in Chickens, China. Emerg. Infect. Dis..

[22] Yu M., Xing L., Chang F., Bao Y., Wang S., He X., Wang J., Wang S., Liu Y., Farooque M. (2019). Genomic Sequence and Pathogenicity of the First Avian Metapneumovirus Subtype B Isolated from Chicken in China. Vet. Microbiol..

[23] Mayahi M., Momtaz H., Jafari R.A., Zamani P. (2017). Detection and Subtyping Avian Metapneumovirus from Turkeys in Iran. Vet. Res. Forum Int. Q. J..

[24] Wang S., Jiang N., Jiang L., Zhuang Q., Chen Q., Hou G., Xiao Z., Zhao R., Li Y., Zhao C. (2022). Establishment and Application of a Quadruple Real-Time RT-PCR for Detecting Avian Metapneumovirus. PLoS ONE.

[25] Cook J.K.A. (2000). Avian Pneumovirus Infections of Turkeys and Chickens. Vet. J..

[26] Eterradossi N., Toquin D., Guittet M., Bennejean G. (1992). Discrepancies in Turkey Rhinotracheitis ELISA Results Using Different Antigens. Vet. Rec..

[27] Mekkes D.R., De Wit J.J. (1998). Comparison of Three Commercial ELISA Kits for the Detection of Turkey Rhinotracheitis Virus Antibodies. Avian Pathol..

[28] Li Y., Yu Z., Jiao S., Liu Y., Ni H., Wang Y. (2020). Development of a Recombinase-Aided Amplification Assay for Rapid and Sensitive Detection of Porcine Circovirus 3. J. Virol. Methods.

[29] Tu F., Yang X., Xu S., Chen D., Zhou L., Ge X., Han J., Zhang Y., Guo X., Yang H. (2021). Development of a Fluorescent Probe-based Real-time Reverse Transcription Recombinase-aided Amplification Assay for the Rapid Detection of Classical Swine Fever Virus. Transbound. Emerg. Dis..

[30] Wang Y., Nie M., Deng H., Lai S., Zhou Y., Sun X., Zhu L., Xu Z. (2022). Establishment of a Reverse Transcription Recombinase-Aided Amplification Detection Method for Porcine Group a Rotavirus. Front. Vet. Sci..

[31] Li X., Shen X., Li M., Qi J., Wang R., Duan Q., Zhang R., Fan T., Bai X., Fan G. (2019). Applicability of Duplex Real Time and Lateral Flow Strip Reverse-Transcription Recombinase Aided Amplification Assays for the Detection of Enterovirus 71 and Coxsackievirus A16. Virol. J..

[32] Wang C., Wang C., Zhang Z., Li X., Zhang T. (2023). Research Note: Application of Reverse-Transcription Recombinase-Aided Amplification-Lateral Flow Dipstick Method in the Detection of Infectious Bursal Disease Virus. Poult. Sci..

[33] Duan X., Ma W., Jiao Z., Tian Y., Ismail R.G., Zhou T., Fan Z. (2022). Reverse Transcription-Recombinase-Aided Amplification and CRISPR/Cas12a-Based Visual Detection of Maize Chlorotic Mottle Virus. Phytopathol. Res..

[34] Rausch F., Tanneberger F., Abd El Wahed A., Truyen U. (2023). Validation of the Efficacy of Air Purifiers Using Molecular Techniques. PLoS ONE.

[35] Wang S., Zhuang Q., Jiang N., Zhang F., Chen Q., Zhao R., Li Y., Yu X., Li J., Hou G. (2023). Reverse Transcription Recombinase-Aided Amplification Assay for Avian Influenza Virus. Virus Genes.

[36] Behboudi S. (2022). Turkey Rhinotracheitis. CABI Compend..

[37] Cook J., Huggins M., Woods M., Orbell S., Mockett A. (1995). Protection Provided by a Commercially Available Vaccine against Different Strains of Turkey Rhinotracheitis Virus. Vet. Rec..

[38] Naylor C.J., Lupini C., Brown P.A. (2010). Charged Amino Acids in the AMPV Fusion Protein Have More Influence on Induced Protection than Deletion of the SH or G Genes. Vaccine.

[39] Hu H., Roth J.P., Estevez C.N., Zsak L., Liu B., Yu Q. (2011). Generation and Evaluation of a Recombinant Newcastle Disease Virus Expressing the Glycoprotein (G) of Avian Metapneumovirus Subgroup C as a Bivalent Vaccine in Turkeys. Vaccine.

[40] Patnayak D.P., Goyal S.M. (2004). Cold-Adapted Strain of Avian Pneumovirus as a Vaccine in One-Day-Old Turkeys and the Effect of Inoculation Routes. Avian Dis..

[41] Rubbenstroth D., Dalgaard T.S., Kothlow S., Juul-Madsen H.R., Rautenschlein S. (2010). Effects of Cyclosporin A Induced T-Lymphocyte Depletion on the Course of Avian Metapneumovirus (aMPV) Infection in Turkeys. Dev. Comp. Immunol..

[42] Cha R.M., Khatri M., Sharma J.M. (2011). Protection against Avian Metapneumovirus Subtype C in Turkeys Immunized via the Respiratory Tract with Inactivated Virus. Vaccine.

[43] Xiao Z., Wang S., Zhao C. (2022). Epidemiological Investigation of Avian Metapneumovirus Insome Areas of China in 2020. Chin. J. Prev. Vet. Med..

[44] Chen W., Fan J., Li Z., Zhang Y., Qin Y., Wu K., Li X., Li Y., Fan S., Zhao M. (2021). Development of Recombinase Aided Amplification Combined with Disposable Nucleic Acid Test Strip for Rapid Detection of Porcine Circovirus Type 2. Front. Vet. Sci..

[45] Xia W., Chen Y., Ding X., Liu X., Lu H., Guo C., Zhang H., Wu Z., Huang J., Fan Z. (2022). Rapid and Visual Detection of Type 2 Porcine Reproductive and Respiratory Syndrome Virus by Real-Time Fluorescence-Based Reverse Transcription Recombinase-Aided Amplification. Viruses.

[46] Wu X., Kong J., Yao Z., Sun H., Liu Y., Wu Z., Liu J., Zhang H., Huang H., Wang J. (2022). A New Rapid and Sensitive Method for Detecting Chicken Infectious Anemia Virus. Front. Microbiol..

[47] Hess M., Huggins M.B., Mudzamiri R., Heincz U. (2004). Avian Metapneumovirus Excretion in Vaccinated and Non-Vaccinated Specified Pathogen Free Laying Chickens. Avian Pathol..

[48] Xu W., Suderman M., Koziuk J., Ojkic D., Berhane Y. (2021). Development of A Recombinant Nucleocapsid Based Indirect ELISA for the Detection of Antibodies to Avian Metapneumovirus Subtypes, A, B, and C. Vet. Immunol. Immunopathol..

[49] Cook J.K.A., Huggins M.B., Orbell S.J., Senne D.A. (1999). Preliminary Antigenic Characterization of an Avian Pneumovirus Isolated from Commercial Turkeys in Colorado, USA. Avian Pathol..

