# Supplementary figures and images for: Establishment and Application Prospect of Reverse Transcriptase Recombinase-Aided Amplification Assay for Subgroup C Avian Metapneumovirus
Source: Vet Sci. 2024 Mar 7;11(3):122. doi: 10.3390/vetsci11030122 (PMC10975772; doi:10.3390/vetsci11030122)

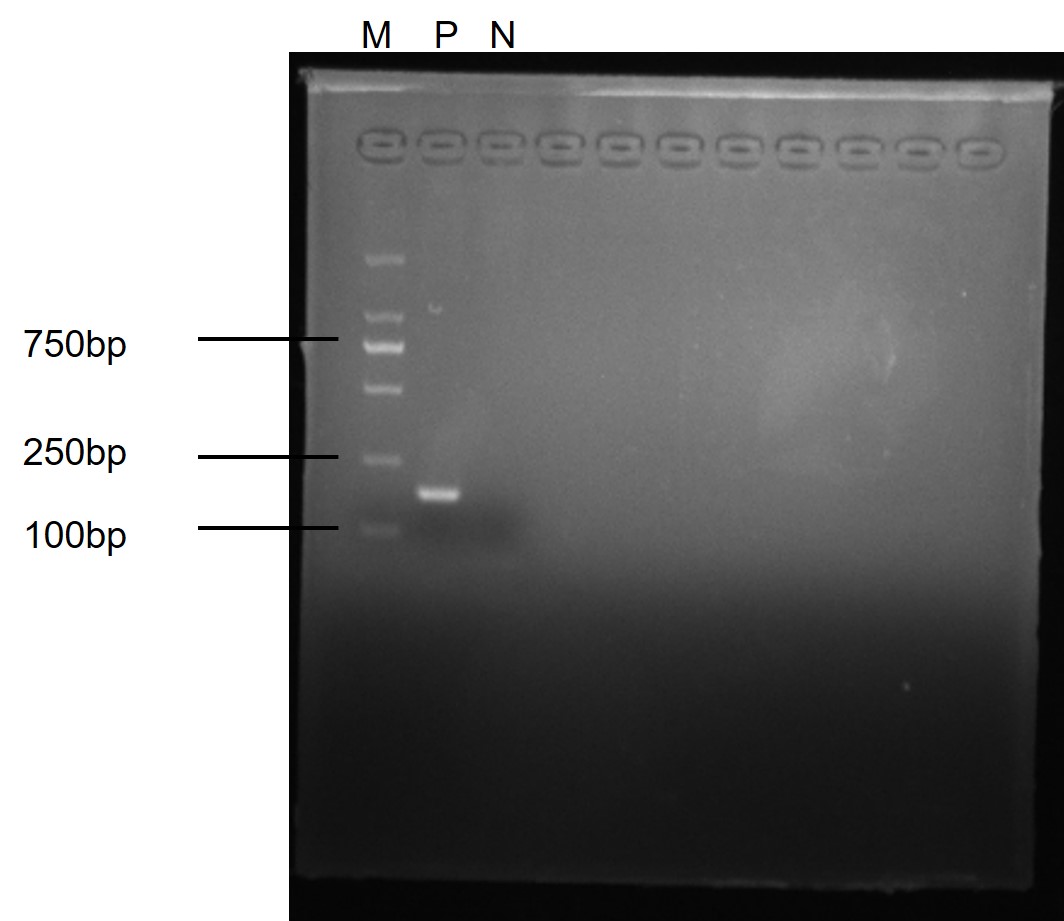

Supplement: Supplementary file 1 [file vetsci-11-00122-s001.zip › vetsci-2829326-supplementary.jpg]
